# Longitudinal Evidence of How Media Audiences Differ in Public Health Perceptions and Behaviors During a Global Pandemic

**DOI:** 10.3389/fpubh.2020.583408

**Published:** 2020-12-02

**Authors:** Thomas Frissen, David De Coninck, Koenraad Matthys, Leen d'Haenens

**Affiliations:** ^1^Faculty of Social Sciences, Institute for Media Studies, KU Leuven, Leuven, Belgium; ^2^Department of Society Studies, Faculty of Arts and Social Sciences, Maastricht University, Maastricht, Netherlands; ^3^Faculty of Social Sciences, Centre for Sociological Research, KU Leuven, Leuven, Belgium

**Keywords:** COVID-19, public health behavior, media exposure, media audiences, longitudinal survey data, public health communication

## Abstract

The current study investigates how public attitudes and perceptions about the COVID-19 pandemic evolve over time and influence self-reported health behaviors (e. g., social distancing). Specific attention was paid to respondents' exposure to different news media channels (public vs. commercial). We used data from a two-wave panel study with a 3-week interval (W1 at the start and W2 at the peak of the pandemic) and a large sample of the adult population in Flanders, Belgium (*n* = 870). The results of mixed ANOVAs indicate that besides a time-effect there was also a significant effect of the different types of news media exposure and respondents' support for protective health measures and behaviors. Whereas, perceived vulnerability to disease, feelings of loneliness, and solidarity were mostly determined by respondents' overall frequency of media exposure, support of governmental measures and self-reported health behaviors were mostly determined by the type of news media exposure. Respondents with a predominantly public/quality news media diet had the highest scores on these variables. A stepwise linear regression analysis with individual's change scores demonstrated that (self-)protective behavior was positively determined by respondents' age, solidarity, and the belief that the measures are necessary, but negatively determined by one's cumulative exposure to commercial/tabloid news media. This longitudinal study provides a new perspective on the role of news media in times of a public health crisis. It offers support for (A) the “double bind hypothesis” (i.e., while news media consumption encourages (self-)isolation, it fosters feelings of loneliness); and (B) the “dual effects hypothesis” (i.e., exposure to commercial/tabloid news media generates different outcomes than exposure to public/quality news media). Affective responses and socio-psychological perceptions are influenced by overall news media exposure, whereas support for the government and its handling of the crisis are mainly determined by one's selection of media channels, whereby audiences of public news media evaluate these outcomes more positively than the audiences of commercial news media channels.

## Introduction

Recently, the coronavirus disease 2019 (COVID-19) has been rapidly expanding across the globe. In order to respond to this pandemic, many countries are combining suppression and mitigation activities aimed at delaying major surges of patients and leveling the demand for hospital beds, while protecting the most vulnerable from infection (1). It is crucial for the public's health that information about these measures is accurately and quickly disseminated throughout the population, especially when considering that threat perceptions of novel viral infections are higher compared to perceptions of common threats like influenza (2). The global scale of the current crisis, and the introduction of measures such as social distancing leads to increased anxiety and stress, which in turn have a detrimental impact on the public's physical and mental health over time, as evidenced by longitudinal studies following other health or societal crises (3, 4). Furthermore, in addition to disseminating and contextualizing information regarding public health measures, it is also important to stimulate public support for these measures, especially given their fundamental impact on daily life. A lack of support for such measures may result in the public not abiding by certain guidelines, which may in turn endanger public health (5).

Currently, traditional news media (e.g., television, radio, newspapers) and social media are the main platforms through which this dissemination of information takes place (6, 7). In fact, traditional media are even believed to have experienced a “revival” during the COVID-19 pandemic, as most people “retrogressed back” to these “established” media environments that provide them with “trustworthy” and verified information or news updates (8). The public's reliance on news media to convey accurate information is especially important during this crisis, with a large share of the population working from or locked down in their homes (3). Two elements of media exposure have been found to affect psychological and physical responses to a community-wide traumatic event: the amount of media exposure, and its content (7).

Concerning the total amount of exposure to the media: Garfin et al. (7) use the Boston Marathon bombings as an example, where they found a “strong positive association between the total amount of exposure to bombing-related media coverage and acute stress symptoms. People who reported the highest media exposure reported higher acute stress than people who were directly exposed to the bombings” (7, 9). These associations accumulate over time: as threats continue to emerge, repeated high levels of media exposure to these kinds of events create a cycle of distress (7, 10).

As for the context of this coverage: studies found that overly sensationalized and tabloidized coverage of traumatic events (e.g., graphic imagery) is related to higher stress levels among the public, even after controlling for the overall amount of media exposure (7). In that regard, it is important to note that not all media types frame stories the same way. An international comparison of media systems showed that commercial media present significantly more sensationalized news than public media (11). Recent Belgian data corroborate this trend. Jacobs et al. found that Belgian public news media are significantly less sensationalist and “tabloidized” than commercial news media in the context of contentious and crisis-related topics (such as immigration) and that the audiences of both news types differ in their attitudes toward the covered topics. In this context, it has been assumed that outcomes of mediated communication are not uniform across all individuals. Instead, media effects tend to vary across different media channels (i.e., “dual effects hypothesis”) (12–14), and exposure to a given news medium means exposure to multiple messages that may be incongruent and exert therefore conflicting and contradictory influences within an individual (i.e., “double bind hypothesis”) (15, 16).

In the current longitudinal study, we aim to test how public fears and attitudes toward public health measures evolve over time during the COVID-19 pandemic (research question (RQ)1) while accounting for one's exposure to different news media channels (public vs. commercial) in Flanders (Belgium) (RQ2). Additionally, we aim to examine how later-stage self-reported health behaviors (e.g., social distancing) are associated with (a) evolutions in public perceptions and attitudes regarding the disease and (b) respondents' accumulative exposure to different news media channels (RQ3).

## Materials and Methods

### Sampling Procedure

We used the data of a two-wave online panel study that was conducted in locked-down Flanders with an interval of 3 weeks. The first wave (W1) took place 3 days after the government installed the first set of restrictive measures in the country, such as social distancing and telecommuting, and ran from March 17, 2020 to March 22, 2020. Additionally, on the first day of the data collection, the government decided to go in full lockdown—i.e., the closing down of all non-essential shops and business and non-essential movements were forbidden. The second wave (W2) data were collected at the peak of the outbreak in Belgium in terms of new cases and COVID-related deaths, and ran from April 6, 2020 to April 18, 2020.

Adults ranging from 18 to 70 years of age from Flanders, the northern, Dutch-speaking region of Belgium, made up the research population. Respondents were recruited through polling agency iVOX. Their large pool of research candidates was contacted by e-mail and the survey was distributed via the agency's survey software. The survey language was Dutch. Prior to filling out the survey, respondents had to accept an informed consent form in which they were briefed about the study's design and approach. Only those respondents who answered all questions were retained in the final sample.

At baseline (W1), 1,000 adults participated (response rate = 32%). Three weeks later, in the follow-up survey (W2), 870 out of the 1,000 respondents who participated in W1 participated again (response rate between W1 and W2 = 87%). Only the respondents of the final sample were used for the analyses of the current study (*n* = 870) and their characteristics can be found in Table 1 (17).

**Table 1:** Sample description of respondents who took part in both W1 and W2 (*n* = 870).

		**Frequency (%)**
Sex	Male	446 (51.3%)
	Female	424 (48.7%)
Age (W1)	18–34	239 (27.5%)
	35–54	257 (29.5%)
	55–70	374 (43%)
Educational attainment (W1)	Secondary education or lower	456 (52.4%)
	Tertiary education	414 (47.6%)
Symptoms of COVID-19 (W1)	Yes	31 (3.6%)
	No	839 (96.4%)
Symptoms of COVID-19 (W2)	Yes	83 (9.5%)
	No	787 (90.5%)
Symptoms of COVID-19 (W1 or W2)	Yes	93 (10.7%)
	No	777 (89.3%)

### Instruments of Measurement

#### Exposure to News Media and Membership of Different Media Audience (W1 and W2)

In order to assess participants' exposure to news media about the COVID-19 pandemic, we gauged the frequency of exposure to eight Flemish news media sources. This was done by asking respondents in both waves to rate how often they had consulted the media sources in the week prior to the survey: (1) public television, (2) public radio, (3) quality newspapers, (4) social media channels of public/quality news media, (5) commercial television, (6) commercial radio, (7) tabloids, and (8) social media channels of commercial/tabloid news media. Examples of each media source were provided (for instance the “VRT” for public television news; “VTM” for commercial television news; “De Standaard” and “De Morgen” for quality press, and “HLN” and “Het Nieuwsblad” for tabloid press). Respondents were asked to respond to each item on a 5-point Likert scale ranging from 1 “never” to 5 “multiple times a day.” Principal component analysis with varimax rotation yielded a two-factor structure: one factor with the public news media and quality press itemsand one with the commercial and tabloid press items. Both components showed reasonable reliability in both W1 and W2 (Cronbach's Alpha for public/quality media sources = 0.60 (W1) and 0.62 (W2); Cronbach's Alpha for commercial/tabloid media sources = 0.60 (W1) and 0.57 (W2). A composite measure for each component was created. A higher score means a higher frequency of exposure to the specific news media sources.

In order to assign each respondent to a unique media audience condition, we used a two-step approach. First, we used a median split on the composite measures to create a high/low categorical variable of both the public/quality news media variable and the commercial/tabloid news media variable. Second, we made a 2x2 matrix with both binary news media variables, which enables the creation of four distinct groups: (1) respondents who scored low on public/quality news media and low on commercial/tabloid news media were assigned to the group “*Low overall news media consumption”* (*n* = 246), (2) respondents who scored high on public/quality news media and high on commercial/tabloid news media were assigned to the group “*High overall news media consumption”* (*n* = 199), (3) respondents who scored high on public/quality news media and low on commercial/tabloid news media were assigned to the group “*Predominant public/quality news media consumption”* (*n* = 195), and (4) respondents who scored high on commercial/tabloid news media and low on public/quality news media were assigned to the group “*Predominant commercial/tabloid news media consumption*” (*n* = 230).

Important to note here is that we use the term “quality news media” in our manuscript in order to connect with the existing body of literature that has followed a similar dichotomy of “quality press” vs. “tabloid press” (or “infotainment”) (12). Other dichotomies that are well-known in the existing journalism literature are “hard news” vs. “soft news” or “high-brow” vs. “low-brow news” (18, 19). Quality press (or “hard news” or “high-brow news,” or originally referred to as “broadsheet press”) is used for news outlets that differ from tabloid press in terms of the format, themes, focus, and style. Generally speaking, the term quality press refers to news that is more serious, more detailed, less emotional and less personal (e.g., The Guardian and The News York Times). Quality is therefore not used as a normative label in this study [cfr. the quality news vs. “fake news”-debate (20)] but as conceptual/analytical label that is derived from the quality vs. tabloid dichotomy in the journalism literature.

#### Perceived Vulnerability to Disease (W1 and W2)

To measure respondents' perceived vulnerability to disease over the course of time we used the 15-item self-report measurement as developed and validated by Duncan et al. (21), in both W1 and W2. Participants were asked to answer each statement on the basis of a 7-point Likert scale ranging from 1 = “strongly disagree” to 7 = “strongly agree.” Six items had to be reversed coded, so that a higher score on the item meant higher perceived vulnerability. This scale contains two subscales: perceived infectability (seven items) and germ aversion (eight items). Perceived infectability assesses one's “beliefs about immunological functioning and personal susceptibility to infectious diseases” [(21), p. 542]. Germ aversion assesses one's “aversive affective responses to situations that connote a relatively high likelihood of pathogen transmission” [(21), p. 542]. Principal component analysis with varimax rotation confirmed this factor structure in the present data. Internal consistency was satisfactory for both subscales in both waves [Cronbach's alpha for perceived infectability = 0.85 (W1) and 0.84 (W2); Cronbach's alpha for germ aversion = 0.69 (W1) and 0.68 (W2)].

#### Socio-Economic and Socio-Psychological Perceptions (W1 and W2)

We included three items in both waves to measure respondents' socio-economic and socio-psychological perceptions of the measures taken by the government to contain the COVID-19 pandemic over time. These were (1) to what extent respondents believed that the measures will result in an economic crisis (perception of economic crisis), (2) whether respondents believed they will experience loneliness in the coming weeks (loneliness), and (3) to what extent respondents are willing to go in quarantine if they feel unwell (solidarity). All items were measured on a 5-point Likert scale ranging from “strongly disagree” to “strongly agree.”

#### Attitudes Toward Public Health Measures (W1 and W2)

We used two items to measure respondents' attitudes toward the public health measures taken by the Belgian government. The first item gauged to what extent respondents believed that the measures taken by the government were necessary to protect the population. The second one measured the extent to which participants believed that the Belgian government was handing the crisis well. For both items, a 5-point Likert scale was used ranging from 1 = “strongly disagree” to 5 = “strongly agree.”

#### (Self)-Protective Behavior (W2 Only)

In contrast to the other variables in this study, (self-)protective behavior was measured only in Wave 2. We asked respondents to self-report on a 5-point Likert-scale (1 = “I don't follow this at all; 5 = “I follow this perfectly”) to what extent they followed the several COVID-19 measures to protect themselves and to prevent propagation of the virus. Four (self-)protective behaviors were assessed: (1) non-essential travel, (2) social distancing (i.e., keeping a distance of 1.5 m), (3) washing hands regularly, and (4) no gatherings of more than two people. Principal component analysis with varimax rotation and parallel analysis indicated that all four items load on one factor. As such, we computed for each respondent a mean score for (self-)protective behaviors with a good reliability (Cronbach's alpha = 0.79).

## Results

**Figure 1:**
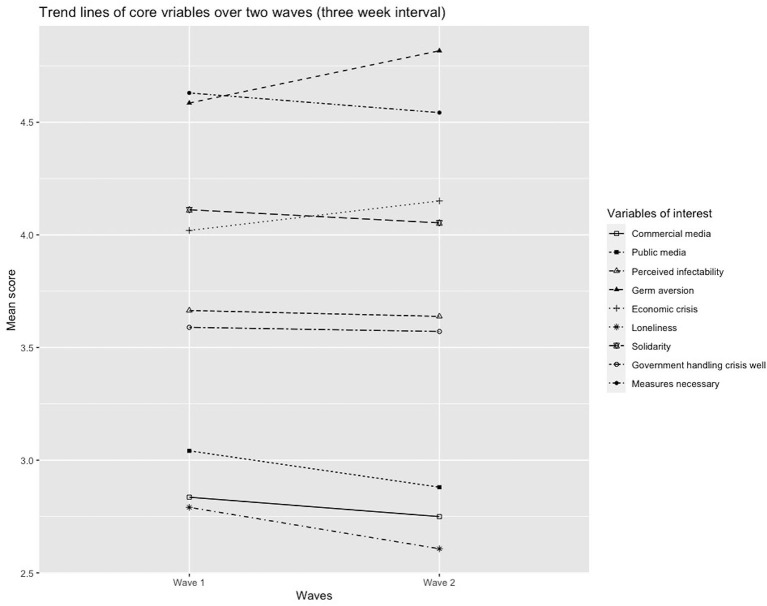
Visualization of the evolution of the core variables over time (3 week interval). All variables were measured on a 5-point scale, except for perceived infectability and germ aversion (7-point scale).

In reference to our first two research questions, we started with an explorative analysis of how the core variables evolved over time. The trendlines in Figure 1 visualize the paths for each variable (see also Appendix). Additionally, we conducted multiple mixed ANOVAs, to analyze the effects of both the time (within groups) and the four different media audiences (between groups) on the dependent variables (see Table 2). We used pairwise comparisons with Bonferroni correction to compare mean scores between both time points and across the four media audiences at W1 and the dependent variables in W2.

**Table 2:** Results of mixed ANOVAs of the core variables.

	**F-test and effects size**							
	**Media audience group**					**Time**		
		**High**	**Low**	**P/Q**	**C/T**		**W1**	**W2**
Perceived infectability	*F*_(3, 866)_ = 3.53, $\eta_{\text{G}}^{2}$ = 0.01^*^	**3.80 (1.15)**	3.53 (1.05)	3.56 (1.00)	3.68 (1.14)	*F*_(1, 866)_ = 1.703, n.s	3.66 (1.10)	3.64 (1.09)
Germ Aversion	*F*_(3, 866)_ = 9.641, $\eta_{\text{G}}^{2}$ = 0.03^***^	**4.99 (1.01)**	4.58 (1.03)	4.80 (0.97)	4.94 (0.94)	*F*_(1, 866)_ = 85.57, $\eta_{\text{G}}^{2}$ = 0.02^***^	4.59 (1.00)	**4.82 (1.00)**
Perceptions of economic crisis	*F*_(3, 866)_ = 2.189, n.s.	4.19 (0.83)	4.07 (0.85)	4.15 (0.74)	4.20 (0.81)	*F*_(1, 866)_ = 19.296, $\eta_{\text{G}}^{2}$ = 0.01^***^	4.02 (0.89)	**4.15 (0.81)**
Loneliness	*F*_(3, 866)_ = 4.780, $\eta_{\text{G}}^{2}$ = 0.01^**^	**2.76 (1.20)**	2.47 (1.26)	2.48 (1.19)	2.73 (1.25)	*F*_(1, 866)_ = 25.623, $\eta_{\text{G}}^{2}$ = 0.01^***^	**2.79 (1.26)**	2.61 (1.24)
Solidarity	*F*_(3, 783)_ = 5.880, $\eta_{\text{G}}^{2}$ = 0.02^***^	**4.25 (0.78)**	3.94 (0.88)	4.12 (0.83)	3.96 (0.93)	*F*_(1, 783)_ = 3.825, n.s	4.11 (0.85)	4.05 (0.87)
Handling well	*F*_(3, 866)_ = 6.648, $\eta_{\text{G}}^{2}$ = 0.02^***^	3.54 (0.96)	3.50 (0.96)	**3.80 (0.78)**	3.48 (0.97)	*F*_(1, 866)_ = 0.51, n.s	3.59(1.01)	3.57 (0.93)
Measures necessary	*F*_(3, 866)_ = 5.556, $\eta_{\text{G}}^{2}$ = 0.02^***^	4.53 (0.72)	4.44 (0.88)	**4.68 (0.54)**	4.54 (0.77)	*F*_(1, 866)_ = 15.247, $\eta_{\text{G}}^{2}$ = 0.04^***^	**4.63 (0.68)**	4.54 (0.75)
(Self-)protective behavior	*F*_(3, 866)_ = 3.756, $\eta_{\text{G}}^{2}$ = 0.013^*^	3.70 (0.77)	3.54 (0.70)	**3.75 (0.50)**	3.63 (0.75)	N.A.		

*The interaction between media group and time was for none of the variables significant. $\eta_{\text{G}}^{2}$ = generalized effect size*;

* *p < 0.05*,

** *p < 0.01*,

*** *p < 0.001, n.s., not significant; P/Q, Public/quality news media group; C/T, Commercial/tabloid news media group. Bold indicates the highest mean score between groups*.

A significant effect for time was found for four variables. Respondents' germ aversion (mean difference = 0.23) and perceptions of an economic crisis (mean difference = 0.13) increased significantly during the lockdown. In contrast, feelings of loneliness (mean difference = −0.18), and the belief that measures are necessary (mean difference = −0.09) declined significantly. In the case of the latter, it cannot go unmentioned that a potential ceiling effect was at play. At baseline, respondents rated the necessity of the restrictive measures taken by the government already relatively high (M_W1_= 4.63). Respondents' exposure to public news media [mean difference = −0.16, *t*_(869)_ = −6.52, *p* < 0.05] and commercial news media [mean difference = −0.09, *t*_(869)_ = −6.52, *p* < 0.05] has decreased over time.

A significant effect of the media audiences was found for nearly all variables, with an exception for participants' economic perceptions [*F*_(3,866)_ = 2.189, *p* = 0.088]. For perceived infectability, the data show that respondents in the high overall media exposure group scored significantly higher (M = 3.8; SD = 1.15), than respondents in the low overall exposure group (M = 3.53, SD = 1.05) and respondents in the predominantly public/quality media group (M = 3.56, SD = 0.99). For germ aversion, respondents in the low overall exposure condition had on average a significantly lower score for germ aversion (M = 4.58, SD = 1.03) than the respondent of all other media audiences (M_high exposure =_ 4.99, SD = 1.01; M_commercial dominant_ = 4.94, SD = 0.94; M_public dominant_ = 4.80, SD = 0.97). Respondents in the high overall media exposure condition scored the highest on perceptions of loneliness (M = 2.76, SD = 1.20). This was significantly lower for the low media exposure condition (M = 2.47, SD = 1.26) and the public/quality news media condition (M = 2.48, SD = 1.19). Interestingly, respondents who chose to consume mainly public/quality news media were also less lonely than those who consumed mainly commercial/tabloid news media (M = 2.73, SD = 1.25). In terms of solidarity, the high overall media exposure group had the highest tendency to go in quarantine if necessary (M = 4.25, SD = 0.78). This was significantly lower for the people who chose for either a commercial-dominant media diet (M = 3.96, SD = 0.93) or just little media exposure in general (M = 3.94, SD = 0.88). In contrast to the latter, also respondents in the public/quality media condition reported a higher degree of solidarity (M = 4.12, SD = 0.83). The results of these four analyses seem to suggest that people's perceptions of vulnerability to disease (i.e., perceived infectability and germ aversion) and socio-psychological outcomes (i.e., loneliness and solidarity) are mainly dependent on respondents' overall frequency of media exposure and to a lesser extent influenced by the specific type of news media source that is consumed.

Interestingly, the data show a different pattern for the two items that gauge respondents' attitudes toward the measures taken by the Belgian government. The audiences of the public/quality news media tend to be the most supportive toward the way in which the government is handling the crisis (M = 3.8, SD = 0.784) compared to all other audience groups (M_high exposure =_ 3.54, SD=.96; M_low exposure_ = 3.50, SD = 0.96; M_commercial dominant_ = 3.48, SD = 0.97). People with a predominantly commercial media diet tend to be the least supportive for how the government is handling the crisis. A similar finding emerges when we compare the belief that the measures taken by the government were necessary across the four audience groups. Respondents with a predominantly public/quality news media diet rate the necessity of the measures significantly higher (M = 4.68, SD = 0.54) than respondents in the high overall exposure group (M = 4.53, SD = 0.72) and in the low overall exposure group (M = 4.44, SD = 0.88). Even though the difference between the public/quality news media group and the commercial/tabloid news media group was not statistically significant, the latter rated the necessity of the measures substantially lower (M = 4.54, SD = 0.77). These findings indicate that in our sample it is mainly the type of the news source (i.e., public/quality vs. commercial/tabloid), that seems to play an important role in terms of governmental support in times of crisis.

To evaluate whether one's (self-)protective behavior was significantly different across the four media audiences, a one-way ANOVA was performed. The analysis shows that respondents' behavior to protect themselves and to prevent the virus from spreading did indeed differ significantly between the media audiences [*F*_(3,866)_ = 3.756, *p* > 0.05]. Respondents in the low overall exposure group were the least willing to behave in a (self-)protective manner (M = 3.54, SD = 0.699). Pairwise comparison using Bonferroni correction showed that this was significantly lower than respondents in the high overall exposure group (M = 3.70, SD = 0.77) and the ones in the public/quality news media group (M = 3.75, SD = 0.498). In fact, respondents who consume a predominantly public/quality news media diet appear to behave most favorable in order to protect themselves and to prevent the virus from spreading.

Finally, in reference to research question three, we conducted a stepwise linear regression model to investigate whether the changes over time affect respondents' (self-) protective behavior at time 2. In order to do this, we calculated an evolution score for each variable. This was done by subtracting the mean of the W1 score from the mean of the W2 score (e.g., evolution in perceived infectability = mean of perceived infectability W2—mean of perceived infectability W1). In all steps, we controlled for sex, age, educational attainment, and whether the respondent had reported to have been suffering from COVID-19 symptoms in either W1 or W2 (see Table 3). In the last block, we also included an interaction between both types of news media exposure in order to control for the potential influence of an overall increase in news media exposure over time.

**Table 3:** Results of a stepwise linear regression with (self-)protective behavior as the criterion and changes over time for the predictor variables (i.e., differences in means).

**Predictor**		**Model 1**	**Model 2**	**Model 3**	**Model 4**	**Model 5**
Socio demographics	Sex	0.06	0.07	0.06	0.06	0.07
	Age	0.27^**^	0.27^**^	0.26^**^	0.27^**^	0.27^**^
	Educational attainment	0.05	0.05	0.05	0.05	0.05
	COVID-19 symptoms	0.03	0.03	0.02	0.02	0.02
Perceived vulnerability to disease	Perceived infectability		−0.06	−0.06	−0.05	−0.05
	Germ aversion		0.06	0.06	0.05	0.05
Socio-economic and socio-psychological perceptions	Solidarity			0.08^*^	0.08^*^	0.08^*^
	Perceptions of economic crisis			0.05	0.05	0.06
	Loneliness			−0.01	−0.01	−0.01
Attitudes toward government and public health measures	Government is handling well				0.03	0.03
	Measures necessary				0.08^*^	0.09^*^
Type of media exposure	Commercial/tabloid news media					−0.08^*^
	Public/quality news media					−0.01
	Overall exposure (i.e., interaction; commercial*public media)					−0.06
			Δ*R^2^* = 0.006	Δ*R^2^* = 0.010^*^	Δ*R^2^* = 0.008^*^	Δ*R^2^* = 0.007
		*R^2^* = 0.074^**^	*R^2^* = 0.081^**^	*R^2^* = 0.090^**^	*R^2^* = 0.098^**^	*R^2^* = 0.106^**^

*Standardized regression weights (β) are presented*.

* *p < 0.05*,

** *p < 0.01*.

In the full model, the predictors enable us to explain 10% of the variance of (self-) protective behavior. Thereby, the strongest determinant is the respondent's age (β = 0.27, *p* < 0.01). Older respondents tend to follow the (self-)protective health measures better than the younger respondents. Furthermore, the analysis also indicates that an increased solidarity over time (β = 0.08, *p* < 0.01), and an increase in the belief that the measures are necessary (β = 0.09, *p* < 0.01) lead to more (self-)protective behavior at time 2. Interestingly, a change in the consumption of commercial/tabloid news media over time was also a significant determinant for respondent's (self-)protective behavior (β = −0.08, *p* < 0.01). More specifically, a higher consumption of commercial media in time 2 in comparison to time 1 is associated with lower levels of (self-)protective behavior. This suggests that respondents who increased their exposure to commercial/tabloid news media over the course of 3 weeks were less likely to follow the public health measures taken by the government.

## Discussion

The dissemination and contextualization of information during a public health crisis such as the COVID-19 pandemic is crucial to mobilize nation-wide support for restrictive public health measures taken by the government and to prevent the crisis from further escalation. Traditional media, both public and commercial, remain the most important channels through which such information is communicated. Traditional media are even believed to have experienced a “revival” during the COVID-19 pandemic, as most people “retrogressed back” to the environments they knew would provide them with “trustworthy” and established information or news updates (8). However, the way in which different traditional media channels (publicly vs. commercially funded) cover a (nation-wide or global) crisis differs significantly and generates different outcomes in public's attitudes and behaviors (11–13). The current study set out to test how (self-)protective behaviors, public perceptions and attitudes, and support toward public health measures taken by the Belgian government evolve over time during the COVID-19 pandemic, while accounting for one's exposure to different news media channels (“dual effects” and “double bind”). Two-wave panel data that were collected over the course of 3 weeks in a locked-down Belgium provided some interesting new insights.

First, in terms of perceived vulnerability to disease, results show that public's germ aversion increased significantly while perceived infectability did not change over time. Apparently, perceived infectability, which refers to one's beliefs of personal immunological functioning and susceptibility, is a rather stable trait that has not affected meaningfully by the COVID-19 pandemic. However, germ aversion, referring to one's affective reactions to situations with a relatively high risk of infectious pathogen transmission, tends to increase when the pandemic expands and even peaks. This finding is meaningful because it indicates that if individuals are confronted with a virus outbreak such as the COVID-19 pandemic, they also have an increasing affective reaction (i.e., repulsion and disgust) toward situations in which disease transmission is a risk (22). Yet, at the same time, their perception of personal susceptibility does not change over time and remains relatively low.

Looking at the news media consumption, the data show that it is predominantly the overall frequency of news media exposure that determines one's perceived infectability and germ aversion and not so much the choice of a specific news media channel. That suggests that one's affective responses to the crisis are in fact most strongly affected by the mere quantity of media messages rather than by the content, the source, or the sender of the messages [cf. mere exposure (23)].

Second, in terms of socio-economic and socio-psychological perceptions toward the heath crisis, the data show that the belief that the COVID-19 pandemic will result in an economic crisis rose substantially over time. Surprisingly, this was the only outcome that did not differ across the four media conditions. As such, the belief that the Belgian economy is headed toward a crisis appeared to be independent of one's quantity and type of news media exposure and increased equally in all groups during the lockdown. One possible explanation for this could be that all news media channels in Belgium equally covered the subject of an economic crisis with messages of crashing stock markets and unprecedented low oil prices because those were *de facto* serious global (macro-) economic realities (24, 25). Yet, previous studies have shown that Belgian public news media devote more attention to news of economic crises than commercial/tabloid news media (26). Another explanation could be that, independent of any form of media exposure, respondents simply started to experience the (macro-)economic consequences of crisis personally at the moment that W2 data were collected. As in many other countries, all Belgian non-essential shops and enterprises, schools, and industry remained closed for nearly a month, and as a result more than a million Belgians became temporarily unemployed (27).

In contrast to perceptions of an economic crisis, levels of solidarity and loneliness dropped over the course of time. Rather paradoxically, this suggests that while the pandemic was peaking in terms of new cases and COVID-19 related deaths—and thus (self-)isolation and solidarity were most needed—the Belgian public was less willing to be solidary [i.e., willingness to go in (self-)quarantine if one felt sick] and felt at the same time less lonely. Future studies should explore this trend more into detail. Could this mean that self-isolation and loneliness are interrelated in the sense that (the fear of) being lonely (i.e., living in solitary) withholds people from being solidary which in turn may threaten personal and public health? Previous studies have indeed indicated that social isolation resulting in loneliness might be better avoided as it induces anxiety and psychological strain and could perhaps lead to COVID-related suicides (28).

This notion brings us to our findings about a so-called “double bind.” Loneliness and solidarity were both most strongly predicted by the overall frequency of news media exposure. More specifically, the audiences in the high exposure to news media condition experienced the highest levels of loneliness and solidarity. This suggests that news media consumers indeed seem to be caught in a dilemma or “double bind”: on the one hand, news media consumption encourages (self-)isolation, on the other hand, it stimulates feelings of loneliness. Also the media channels are caught in this dilemma: if the they wish to prevent feelings of loneliness, they have to advocate against self-isolation. This is also true the other way around: if they wish to promote self-isolation, they have to accept that their audiences feel lonely. Messages of solidarity and harmony indeed permeated the news media. Nearly all news media channels covered for example—with audiovisual materials—the “#applausvoordezorg”-movement, whereby every night at 8 p.m., many Belgian citizens left their homes to applaud for medical staff (29, 30). However, recent studies investigating the media-loneliness nexus, found that increased exposure to similar messages of harmony on social media are in fact related to one's feelings of loneliness (31). Clearly, more work is needed in order to understand whether such messages of harmony and solidarity in traditional media also affect solidarity and loneliness in a double bind during a global pandemic.

Third, concerning attitudes toward the government and the public health measures: over the course of time and independent of one's media exposure, the belief that the government was handling the crisis well, remained stable. Both in W1 and W2 the Belgian government received relatively high levels of support. However, the belief that the restrictive measures were necessary to prevent the virus from spreading declined substantially. In only 3 weeks the average score for this indicator shifted from principally approval to principally disapproval. Future research should consider multiple-wave and cross-national study designs to test whether this trend continues over a longer period of time and whether it is unique to the Belgian context or rather a globally observed phenomenon.

Interestingly, participants in the predominantly public/quality news media group rated both indicators (i.e., whether the government is handling the crisis well and whether the measures are necessary) higher than those in all other media conditions. This points in the direction that the audiences of public/quality media channels—which are at least partly state-funded—are more in favor of the government and the actions they are taking to curtail the pandemic than audiences of commercial/tabloid media and overall news media consumers. While this finding offers support for the “dual effects hypothesis,” it provokes two potential follow-up questions: (1) to what extent do public/quality news media report more positively about the government than commercial/tabloid media, and (2) Are commercial/tabloid media more critical about the government? Future content analysis of the news media in times of COVID-19 should shed more light on these questions. Additionally, it raises the question of whether the audiences of public and commercial media perhaps differ in terms of trust/distrust in the government. It is not unimaginable that people with lower trust in the government would actually avoid public media channels and rather prefer commercial media because of their actual independence (32). This could then explain why commercial media audiences would be less in favor of the government's decisions, given the known link between trust in the government and support for policy [e.g., (33)]. Future studies should explore this strand more concretely. Is distrust in the government indeed associated with distrust in public news media specifically and does this influence the willingness to support necessary public health policies?

Fourth, in line with the “dual effects hypothesis,” the data showed that (self-)protective behaviors differ significantly across the four media audiences, with respondents in the public/quality media condition reporting the highest levels of (self-)protective behavior. Respondents who consume a predominantly public/quality news media diet appear to behave most favorable in order to protect themselves and to prevent the virus from spreading. In line with this finding, a linear regression model indicated that an increased, cumulative exposure to commercial/tabloid news media during the lockdown predicted lower levels of (self-)protective behavior. A future content analysis of the different news media channels seems necessary in order to explain these findings. Perhaps, the persuasiveness and frames used to stimulate such behaviors differ in both media channels. Indeed, it has been argued that news reporting of a sensationalist and tabloidized nature could undermine democratic outcomes, such as following up on nation-wide restrictive health behaviors (12). From a more media theoretical perspective, the question should be asked whether the commercial/tabloid news media do enough to stimulate their audiences to follow the measures, thereby taking into account the fact that the restrictive public health behaviors are *de facto* conflicting with the potential commercial interests of the sponsors of the commercial media. At the same time, it could be possible that both audiences differ in terms of trust in the government and established institutions (such as the public service media) and therefore differ to the extent to which they would follow their behavioral guidelines. As such, a more sociological study is needed in order to assess whether audiences differ in terms of trust in the government, establishment, and institutions.

Lastly, in summarizing the discussion above, it is of essential importance to discuss the main news media trends that came to surface in the current study. The data clearly demonstrate that news media exposure affects public health outcomes in times of crisis in two very distinct ways. Affective responses such as people's perceptions of vulnerability to disease and socio-psychological outcomes are mainly dependent on respondents' overall frequency of media exposure. In contrast, attitudinal and (self-reported) behavioral outcomes, such as governmental support and (self-)protective behavior are predominantly determined by the channel through which one gets the information and the news (i.e., public/quality vs. commercial/tabloid). Whether this is an effect of the actual media contents or whether this is rather caused by a person's attitude-congruent media selectivity [cfr. selective exposure; reinforcing spirals (34)] remains to be investigated. Similarly, important questions concerning the underlying personal characteristics of the media audiences remain unanswered. For example, who are exactly the commercial/tabloid news media consumers and to what extent do they differ from public/quality news media audiences in terms of social and personality characteristics? Nevertheless, this study offers support for the “dual effects hypothesis,” stating that exposure to different news media channels may generate different outcomes. Furthermore, this study provides new perspectives on the ways in which media audiences are affected by a pandemic or health emergency and to what extent they differ in public health perceptions, attitudes, and behaviors.

The main contribution of this study lies in the differential approach to news media audiences. Both the public health literature as well as the media effects literature seem to be far too often preoccupied with studying the effects of overall frequency of media exposure, without considering the multidimensional nature of the news media and thus the subtle—though meaningful—effects of different lingo, topics, frames, and contents across different news media channels. Future studies are encouraged to combine (automated) content analyses (e.g., topic modeling) of different media channels (publicly vs. commercially funded) with a longitudinal survey design in order to explore this direction more in detail.

## Data Availability Statement

The raw data supporting the conclusions of this article will be made available by the authors, without undue reservation.

## Ethics Statement

Ethical review and approval was not required for the study on human participants in accordance with the local legislation and institutional requirements. The patients/participants provided their written informed consent to participate in this study.

## Author Contributions

TF designed the study, conducted the analyses, wrote the methods, results, and discussion sections. DD co-designed the study and developed the introduction section. KM and Ld'H initiated the study, collected the data, and revised earlier versions of the manuscript. All authors contributed to the article and approved the submitted version.

## Conflict of Interest

The authors declare that the research was conducted in the absence of any commercial or financial relationships that could be construed as a potential conflict of interest.

## Appendix

**Table A1:** Pearson correlations between the change scores (i.e., differences between the mean in W1 and W2) of the core variables.

**Variable**	***M***	***SD***	**1**	**2**	**3**	**4**	**5**	**6**	**7**	**8**
1. Perceived infectability	–0.03	0.66								
2. Germ aversion	0.23	0.74	0.14^**^							
3. Perceptions of economic crisis	0.13	0.87	0.02	0.06						
4. Loneliness	–0.18	1.08	–0.03	–0.03	0.08^*^					
5. Solidarity	–0.06	0.84	–0.01	0.08^*^	0.06	0.07				
6. Handling well	–0.02	0.88	–0.01	–0.01	–0.05	–0.07^*^	0.00			
7. Measures necessary	–0.09	0.67	0.02	0.06	–0.01	–0.02	0.01	0.14^**^		
8. Public media/ quality press	–0.16	0.73	0.01	0.07^*^	–0.02	0.01	–0.04	0.02	0.03	
9. Commercial media/ tabloid press	–0.09	0.72	0.03	0.03	0.04	–0.03	–0.02	0.03	0.05	0.40^**^

*M and SD are used to represent mean and standard deviation, respectively*.

* *p < 0.05*.

** *p < 0.01*.
